# LncRNA-Six1 Encodes a Micropeptide to Activate *Six1* in *Cis* and Is Involved in Cell Proliferation and Muscle Growth

**DOI:** 10.3389/fphys.2017.00230

**Published:** 2017-04-20

**Authors:** Bolin Cai, Zhenhui Li, Manting Ma, Zhijun Wang, Peigong Han, Bahareldin A. Abdalla, Qinghua Nie, Xiquan Zhang

**Affiliations:** ^1^Department of Animal Genetics, Breeding and Reproduction, College of Animal Science, South China Agricultural UniversityGuangzhou, China; ^2^Guangdong Provincial Key Lab of Agro-Animal Genomics and Molecular Breeding, and Key Laboratory of Chicken Genetics, Breeding and Reproduction, Ministry of AgricultureGuangzhou, China; ^3^National-Local Joint Engineering Research Center for Livestock BreedingGuangzhou, China

**Keywords:** lncRNA-Six1, *cis*-acting, micropeptide, *Six1*, muscle growth

## Abstract

Long non-coding RNAs (lncRNAs) play important roles in epigenetic regulation of skeletal muscle development. In our previous RNA-seq study (accession number GSE58755), we found that lncRNA-Six1 is an lncRNA that is differentially expressed between White Recessive Rock (WRR) and Xinghua (XH) chicken. In this study, we have further demonstrated that lncRNA-Six1 is located 432 bp upstream of the gene encoding the protein Six homeobox 1 (*Six1*). A dual-luciferase reporter assay identified that lncRNA-Six1 overlaps the *Six1* proximal promoter. In lncRNA-Six1, a micropeptide of about 7.26 kDa was found to play an important role in the lncRNA-Six1 *in cis* activity. Overexpression of lncRNA-Six1 promoted the mRNA and protein expression level of the *Six1* gene, while knockdown of lncRNA-Six1 inhibited *Six1* expression. Moreover, tissue expression profiles showed that both the lncRNA-Six1 and the *Six1* mRNA were highly expressed in chicken breast tissue. LncRNA-Six1 overexpression promoted cell proliferation and induced cell division. Conversely, its loss of function inhibited cell proliferation and reduced cell viability. Similar effects were observed after overexpression or knockdown of the *Six1* gene. In addition, overexpression or knockdown of *Six1* promoted or inhibited, respectively, the expression levels of muscle-growth-related genes, such as *MYOG, MYHC, MYOD, IGF1R*, and *INSR*. Taken together, these data demonstrate that lncRNA-Six1 carries out *cis*-acting regulation of the protein-encoding *Six1* gene, and encodes a micropeptide to activate *Six1* gene, thus promoting cell proliferation and being involved in muscle growth.

## Introduction

Meat production from chicken muscle is one of the most important factors in determining the economic value of poultry. Numerous processes regulate muscle growth, including genetics, environment, and nutrition, but genetics play the least understood of these critical roles (Scanes et al., 1984).

Some non-coding RNAs are regulators of muscle growth and development, but are not strictly subject to normal genetic analyses (Kallen et al., 2013; Mousavi et al., 2013; Luo et al., 2014). Therefore, these RNAs have been underrepresented in genetic studies, especially those concerning chicken muscle development. Protein-encoding genes only account for a small portion (2%) of the genome, and yet 70 to 90% of the genome is transcribed at some point during development. The result is a large long non-coding RNA (lncRNA) transcriptome in mammals (Lee, 2012).

LncRNAs have been found both in the nucleus and in the cytoplasm, with sizes ranging from 200 bp to more than 100 kb (Cabili et al., 2011; Djebali et al., 2012). LncRNAs are involved in diverse aspects of cell biology and regulation, such as genomic imprinting, X chromosome inactivation, chromatin modifications, nuclear trafficking, transcriptional interference, and activation. These RNAs can regulate gene expression via epigenetic alterations, as well as transcriptional and post-transcriptional processing (Kino et al., 2010; Lee and Bartolomei, 2013; Zhao et al., 2013). LncRNA is an important regulatory factor for biology, but little is known about lncRNA in chicken research. Using next generation sequencing, 281 new intergenic lncRNAs were first systematically identified in chicken embryo skeletal muscle. Further analysis found that these lncRNAs were less conserved than protein-encoding genes, but slightly more conserved than random non-coding sequences (Li et al., 2012). In another study, 1,336 lncRNAs were differentially expressed in the preadipocytes of Jinghai Yellow chicken at different stages, for which the number of differentially expressed genes decreased with differentiation, demonstrating that the early stage might be most important for chicken preadipocyte differentiation (Zhang et al., 2017). LncRNA-αGT is a nuclear, non-coding transcript involved in the stage-specific activation of the chicken adult αD globin gene. Research has shown that lncRNA-αGT is required for full activation of the adult αD gene and maintenance of transcriptionally active chromatin, and could maintain adult α-globin gene expression by promoting an active chromatin structure (Arriaga-Canon et al., 2014). Although, more and more lncRNAs have been found by high-throughput sequencing, the mechanism of lncRNA regulation involved in muscle growth in chicken is still poorly understood.

*Six1* (Sine oculis homeobox 1) is an *SO* (Sine oculis) ortholog in vertebrates and is a member of the Sine oculis homeobox family of highly conserved transcription factors. *Six1* was first identified as a regulator of *Drosophila* visual system development (Cheyette et al., 1994). Subsequently, a role for *Six1* in vertebrate development of sensory systems, skeletal muscle, craniofacial organs, and kidneys has been found (Xu et al., 2003; Stierwald et al., 2004; Nonomura et al., 2010; Gordon et al., 2012; Sato et al., 2012). *Six1* is expressed in numerous tissues and especially in skeletal muscle (Boucher et al., 1996; Wu et al., 2011; Wang et al., 2014). This gene regulates skeletal muscle development and transformation of muscle fiber types throughout the embryonic to postnatal stages (Ozaki et al., 2001; Laclef et al., 2003; Grifone et al., 2004, 2005; Hetzler et al., 2014; O'Brien et al., 2014).

Some lncRNAs regulate gene expression primarily *in cis*, where both the regulatory RNA and the target gene are transcribed from the same locus (Guil and Esteller, 2012). WRR is a broiler chicken with a fast growth rate, which exhibits a different growth performance from the XH chicken (a Chinese native breed with a slow growth rate) at 7 weeks of age (Ouyang et al., 2015). In our previous study, two chicken breeds, WRR and XH, of 7 weeks in age were used for RNA-seq analysis, and a large population of lncRNAs that were differentially expressed between WRR and XH chickens were identified. This was suggestive that these lncRNAs might affect chicken growth. Both *Six1* and lncRNA-Six1 are differentially expressed between WRR and XH chicken, and lncRNA-Six1 is upstream of *Six1* in the chicken genome.

To study the role of lncRNA-Six1 in chicken skeletal muscle growth, we analyzed its molecular function and expression level, and performed an analysis of *Six1* promoter activity to identify the relationship between lncRNA-Six1 and *Six1*. Additionally, biological function and tissue expression profiles were also analyzed for *Six1*.

## Materials and methods

### Ethics statement

All animal experiments were carried out in strict accordance with the regulations for the Administration of Laboratory Animals of Guangdong Province. These experiments were handled in compliance with, and were approved by, the Institutional Animal Care and Use Committee at the South China Agricultural University (Guangzhou, China) with approval number SCAU#0011. All efforts were made to minimize animal suffering.

### Animals and tissue collection

Six 7-week-old XH female chickens were obtained from the Avian Farm of the South China Agriculture University (Guangzhou, China). Chickens were euthanized, and organs and tissues were collected after rapid dissection, then immediately frozen in liquid nitrogen and stored at −80°C. The samples included the cerebrum, cerebellum, hypothalamus, pituitary, heart, liver, spleen, lung, kidney, muscular stomach, glandular stomach, breast muscle, leg muscle, abdominal fat, and subcutaneous fat.

### RNA isolation, complementary DNA (cDNA) synthesis, and real-time (RT) PCR analysis

Total RNA was extracted from each tissue using Trizol reagent (TaKaRa, Japan) following the manufacturer's protocol. The integrity and concentration of all obtained RNA samples were assayed by 1.5% agarose gel electrophoresis, and evaluated by ultraviolet spectroscopy using the 260/280 nm ratio.

A PARIS Kit (Ambion, Life Technologies, USA) was used to harvest the cytoplasmic and nuclear cell lysates. Fresh cultured cells were placed on ice, after being collected and washed once in PBS. Subsequently, cells were resuspended in 500 μL ice-cold Cell Fractionation Buffer and incubated on ice for 10 min. After centrifugation for 5 min at 4°C and 500 × g, the cytoplasmic fraction was carefully aspirated away from the nuclear pellet; thereafter, the nuclear pellet was washed in ice-cold Cell Fractionation Buffer and lysed in Cell Disruption Buffer. Samples were split for RNA isolation, following the manufacturer's protocol.

The PrimeScript RT Reagent Kit with gDNA Eraser (Perfect Real Time) (TaKaRa, Japan) was used to synthesize cDNA. The iTaq Universal SYBR Green Supermix Kit (Toyobo, Japan) was used for cDNA quantification, according to the manufacturer's protocol, in a BioRad CFX96 Real-Time Detection instrument. The chicken β*-actin* gene was used as an internal control. Data analysis was carried out using the comparative 2^−ΔΔCT^ method (Livak and Schmittgen, 2001).

### Primers, small interfering RNAs (siRNAs), and antisense oligonucleotides (ASOs)

Primers were designed using Premier Primer 5.0 software (Premier Biosoft International, Palo Alto, CA, USA) or OLIGO Primer Analysis Software Version 7 (Molecular Biology Insights, USA), and synthesized by Sangon Biotech (Shanghai, China). The major primers used in this study are listed in Table 1. Of these primers, lncRNA-Six1 5′ RACE-outer, lncRNA-Six1 5′ RACE-inner and lncRNA-Six1-3′ RACE were used to clone the full-length of chicken lncRNA-Six1. The other 7 primers (lncRNA-Six1-ORF-1, lncRNA-Six1-ORF-2, lncRNA-Six1-ORF-3, lncRNA-Six1-ORF-4, lncRNA-Six1-ORF-5, lncRNA-Six1-ORF-6, and lncRNA-Six1-ORF-7) were used for the open reading frame (ORF) amplification of lncRNA-Six1, and pSDS-β-actin was used as a positive control. Moreover, primers pSDS-lncRNA-Six1 and pSDS-Six1 were used to amplify the coding sequences, and then construct the overexpression vector. The last 4 primers (pGL3-basic-P1, pGL3-basic-P2, pGL3-basic-P3, and pGL3-basic-P4) were used for the dual-luciferase reporter assay for the promoter region of *Six1*. Primers for RT-quantitative PCR (qPCR) are shown in Table 2. The siRNAs used for the knockdown of *Six1* were synthesized by Guangzhou RiboBio (Guangzhou, China) and are listed in Table 3. LncRNA-Six1 is an RNA molecule present in the cytoplasm and nucleus. The siRNA and ASO that were used for the specific knockdown of lncRNA-Six1 in the cytoplasm and nucleus, respectively, were designed and synthesized by Guangzhou RiboBio (Guangzhou, China), and are listed in Table 3.

**Table 1:** **Primers used for RACE PCR and vector construction**.

**Primer name**	**Forward & reverse**	**Primer sequence (5′–3′)**	**Annealing temperature (°C)**	**Product length (bp)**
lncRNA-Six1-3′RACE		GCCGCCGGCTTTTCTCTTCCCCCTTC	Step down	
lncRNA-Six1-5′RACE-outer		CTAAGGGGTGAAGAACGCAAAGCGGGGC	Step down	
lncRNA-Six1-5′RACE-inner		CGTTACCCGCTGCTTTTCCTACA	63	
lncRNA-Six1-ORF-1	F	GGGG**GGTCTCTAGTG**ATGGGAAGAAAACGCGTGGGA	55	114
	R	GCCG**GGTCTCGTGGG**TCAATGAGGTTTGTGTTGGAGA		
lncRNA-Six1-ORF-2	F	GGGG**GGTCTCTAGTG**ATGGGAAGAAAACGCGTGGGA	55	225
	R	GCCG**GGTCTCGTGGG**TTAAAGTGCGGGTTTCTGGAGAG		
lncRNA-Six1-ORF-3	F	GGGG**GGTCTCTAGTG**ATGGGGCGGTTTTGGGTTTCG	55	195
	R	GCCG**GGTCTCGTGGG**CTATGTGTTTTTAAAGTGCGGGT		
lncRNA-Six1-ORF-4	F	GGGG**GGTCTCTAGTG**ATGGAAGGGAAAATTAGCAGAC	55	342
	R	GCCG**GGTCTCGTGGG**TTATTCCGCCGTGAGAGCTC		
lncRNA-Six1-ORF-5	F	GGGG**GGTCTCTAGTG**ATGGGGGCAGCGGGAGCAC	55	243
	R	GCCG**GGTCTCGTGGG**TTATACGCGCTGAGATAAGCGG		
lncRNA-Six1-ORF-6	F	GGGG**GGTCTCTAGTG**ATGAGGATTTGGGCGAATGAA	55	258
	R	GCCG**GGTCTCGTGGG**TCACTCCAGGCGTGAATGAATA		
lncRNA-Six1-ORF-7	F	GGGG**GGTCTCTAGTG**TGAGGATTTGGGCGAATG	55	333
	R	GCCG**GGTCTCGTGGG**TCATATACTTTCATTTCTTTCT		
pSDS-β-actin	F	GGGG**GGTCTCTAGTG**ATGGATGATGATATTGCTGC	58	1,548
	R	GCCG**GGTCTCGTGGG**AAAAGACACTTGTTGGGTTAC		
pSDS-Six1	F	GGGG**GGTCTCTAGTG**ATGTCGATGCTGCCGTCGTTCG	61	935
	R	GCCG**GGTCTCGTGGG**CGTTCAGGATCACCGTCCGCTA		
pSDS-lncRNA-Six1	F	GGGG**GGTCTCTAGTG**GCCTGCTCCTGCAGCCCC	60	2,380
	R	GCCG**GGTCTCGTGGG**TTTTTTTCCTTTTTTCCTTTTT		
pGL3-basic-P1	F	CCG**CTCGAG**TTTCTCGTCTGAATTTATGGG	57	2,463
	R	CCC**AAGCTT**GCTCTCCAGGATCTTGTAGAG		
pGL3-basic-P2	F	CCG**CTCGAG**GCAACATCACAAGCACTAAAA	57	1,811
	R	CCC**AAGCTT**GCTCTCCAGGATCTTGTAGAG		
pGL3-basic-P3	F	CCG**CTCGAG**ATAAACGCTGCCATAAAGAC	57	1,345
	R	CCC**AAGCTT**GCTCTCCAGGATCTTGTAGAG		
pGL3-basic-P4	F	CCG**CTCGAG**ATGAAAAGGTACCAAACGAA	57	711
	R	CCC**AAGCTT**GCTCTCCAGGATCTTGTAGAG		

*Sequences in bold represent the enzyme cutting sites*.

**Table 2:** **RT-qPCR primers**.

**Primer name**	**Forward & reverse**	**Primer sequence (5′–3′)**	**Annealing temperature (°C)**	**Product length (bp)**
Six1	F	GTCAGCAACTGGTTCAAGA	59	197
	R	AGGAGGACCGAGTTCTGAT		
lncRNA-Six1	F	CTCGTGAGAAGGGGGCAAAA	56	184
	R	GAAGGCGAAACCCAAAACCG		
Micropeptide	F	ATCGGCAGCGTTCTGTTAC	59	116
	R	TTAAAGTGCGGGTTTCTGG		
Six1 promoter region	F	CCGCTTTGCGTTCTTCAC	59	103
	R	CCTTTATTACAATGCGCTCTTC		
β-actin	F	GATATTGCTGCGCTCGTTG	50–65	194
	R	TTCAGGGTCAGGATACCTCTTT		

**Table 3:** **siRNAs and ASO used for RNA interference**.

**Fragment name**	**Sequence (5′–3′)**
si-gga-Six1	GGGAGAACACGGAGAACAA
si-gga-lncRNA-Six1	GCTTATCTCAGCGCGTATA
ASO-gga-lncRNA-Six1	TTAGCAGACACGCGGTCACG

### Rapid-amplification of cDNA ends (RACE)

The partial lncRNA-Six1 sequence was obtained from our previous lncRNA-seq data (accession number GSE58755). RACE PCR was performed to obtain the full-length sequence of the lncRNA-Six1. Total RNA from breast muscle tissue was used as the template for nested-PCR reactions using a SMARTer RACE cDNA Amplification Kit (Clontech, Osaka, Japan), following the manufacturer's instructions. The products of the RACE PCR were cloned into the pJET 1.2/blunt cloning vector (CloneJET PCR Cloning Kit; Fermentas, Glen Burnie, MD, USA) and sequenced by Sangon Biotech (Shanghai, China).

### Plasmid construction

For lncRNA-Six1 overexpression plasmid construction, the full-length sequence of lncRNA-Six1 was amplified by PCR, and cloned into the expression plasmid pSDS-20218 (SiDanSai, Shanghai, China) by using BsaI restriction enzyme. *Six1* overexpression constructs were generated by amplifying the *Six1* coding sequence, which was then subcloned into the overexpression plasmid vector, pSDS-20218 (SiDanSai, Shanghai, China). Seven ORFs of lncRNA-Six1 were also amplified and cloned into pSDS-20218 (SiDanSai, Shanghai, China). A region from the *GFP* gene was amplified and cloned into pSDS-20218 as a negative control, and was named pSDS-Control.

Luciferase reporter vectors including different sized *Six1* promoter fragments were constructed from the chicken genome using XhoI and HindIII restriction sites. The PCR products were excised with XhoI and HindIII restriction endonucleases and ligated into plasmid vector pGL3-Basic (Promega, Wisconsin, USA). The recombinant constructs were named pGL3-basic-P1 (−2247/+216), pGL3-basic-P2 (−1595/+216), pGL3-basic-P3 (−1129/+216), and pGL3-basic-P4 (−495/+216). These were numbered relative to the first base of the initiation codon of the *Six1* gene.

### Cell culture and transfection

DF-1 cells were cultured in DMEM (Gibco, USA) supplemented with 10% (v/v) fetal bovine serum (Hyclone, USA) and 0.2% penicillin/streptomycin (Invitrogen, USA). QM-7 cells were cultured in high-glucose M199 medium (Gibco, USA) with 10% fetal bovine serum, 10% tryptose phosphate broth solution (Sigma, USA), and 0.2% penicillin/streptomycin. All cells were cultured at 37°C in a 5% CO_2_, humidified atmosphere. In this study, DF-1 cells were used to study the function of lncRNA-Six1, lncRNA-Six1-ORF-2, and *Six1* in chicken genome. For QM-7 cells, were used to study the influence of *Six1* on muscle growth.

All transient transfections used Lipofectamine 3000 reagent (Invitrogen, USA) following the manufacturer's protocol. The concentrations used for plasmid transfections were as follows: 6-well plate, 2.5 μg/well; 12-well plate, 1 μg/well; 24-well plate, 0.5 μg/well; 48-well plate, 0.25 μg/well; 96-well plate, 0.1 μg/well. For siRNAs and ASO, a concentration of 100 nM were used. si-lncRNA-Six1 and ASO-lncRNA-Six1 were co-transfected for interference with lncRNA-Six1, and named as si-ASO-lncRNA-Six1 in this study. The PRL-TK vector (Promega, USA) was used as an internal control plasmid for *Renilla* luciferase expression and was co-transfected with the pGL3 luciferase reporter vectors. pSDS-Control and pGL3 basic were used as the empty vector control plasmids. In this study, except for Cell Counting Kit (CCK)-8 assays, all cells were harvested or analyzed after 48 h transfection.

### Dual-luciferase reporter assay

The recombinant plasmids (pGL3-basic-P1, pGL3-basic-P2, pGL3-basic-P3, and pGL3-basic-P4), used to analyze the *Six1* promoter region, were co-transfected with pRL-TK in DF-1 cells. The pGL3-basic was also co-transfected with pRL-TK as a control. Firefly and *Renilla* luciferase activities were measured at 48 h post-transfection using a Dual-GLO Luciferase Assay System Kit (Promega, USA), following the manufacturer's instructions. Luminescence was measured using a Fluorescence/Multi-Detection Microplate Reader (BioTek, USA) and firefly luciferase activities were normalized to *Renilla* luminescence in each well.

### Western blotting assay

DF-1 cellular proteins were extracted using radioimmunoprecipitation assay (RIPA) buffer with phenylmethanesulfonyl fluoride protease inhibitor. Western blot analysis was performed as previously reported (Feng et al., 2016). The antibodies used for Western blots were as follows: Flag tag polyclonal antibody (20543-1-AP; Proteintech, USA; 1:1,000), goat anti-rabbit IgG (H&L)-HRP (BS13278; Bioworld, USA; 1:5,000), rabbit anti-Six1 (sc-9127; Santa Cruz, USA; 1:100), myogenin antibody (orb6492; Biorbyt, UK; 1:100), B103 (DHSB, USA; 0.5 μg/ml), goat anti-rabbit IgG-HRP (BA1054; Boster, China; 1:5,000), peroxidase-goat anti-mouse IgG (BA1051; Boster, China; 1:2,500), and HRP-conjugated monoclonal mouse anti-glyceraldehyde-3-phosphate dehydrogenase (GAPDH) (KC-5G5; Kangchen, China; 1:10,000).

### 5-Ethynyl-2′-deoxyuridine (EdU) assays

At 48 h after transfection, DF-1 cells were incubated at 37°C for 2 h in the presence of 50 μM EdU (RiboBio, China). The cells were then fixed in 4% paraformaldehyde for 30 min and neutralized using 2 mg/mL glycine solution. The cells were permeabilized by adding 0.5% Triton X-100. A solution containing EdU (Apollo Reaction Cocktail; RiboBio, China) was added and the cells were incubated at room temperature for 30 min. The nuclear stain Hoechst 33342 was then added and incubation was continued for another 30 min. The number of EdU-stained cells was determined using images of three randomly selected fields obtained with a fluorescence microscope (TE2000-U; Nikon, Japan).

### Flow cytometry analysis of the cell cycle

Flow cytometry analysis was performed on a BD AccuriC6 flow cytometer (BD Biosciences, USA) and data was processed using FlowJo7.6 software. The cell cultures in growth media were collected after a 48 h transfection and fixed in 70% ethanol overnight at −20°C. Propidium iodide (Sigma, USA) (50 μg/mL) containing 10 μg/mL RNase A (Takara, USA) was added and incubated for 30 min at 37°C in the dark and then the samples were sent for detection.

### CCK-8 assays

For the cell growth assays, cells were seeded in a 96-well plate. After being transfected with (a) pSDS-lncRNA-Six1, (b) si-ASO-lncRNA-Six1, (c) pSDS-Six1, or (d) si-Six1, the proliferation of the cell culture was monitored at 24 h, 48 h, 72 h, and 96 h using the TransDetect CCK (TransGen Biotech, Beijing, China). Every 24 h, 10 μL of CCK solution was added and the absorbance at 450 nm was measured using a Model 680 Microplate Reader (Bio-Rad) after a 1 h incubation.

### Scratch-wound assay

DF-1 cells were seeded in 12-well plates. After a density of 50% confluence was reached, cells were transfected. Subsequently, a linear wound was generated by scratching the monolayer of cells with a 1 mL pipette tip, when the cells reached a density of 100% confluence. The proliferation and migration of cells were captured at 0, 24, 48, and 72 h by using a microscope (TE2000-U; Nikon, Japan). The width of the scratches was measured by Nano Measurer software. The formula to calculate the cell migration rate was as follows: (W0h – W × h) × 100%/ W0h, where W0h represents the mean wound width at 0 h, and W × h represents the mean wound width at 24, 48, and 72 h.

### Chromatin immunoprecipitation assay

After transfection for 48 h, cells were fixed in 1% formaldehyde for 10 min at 37°C, and quenched with 125 mM glycine for 5 min. Samples were then washed twice with cold PBS and placed in SDS lysis buffer. After sonication process [(5 s pulse + 5 s interval) × 25% of maximum power, for 14 times], chromatin was immunoprecipitated with the DYKDDDDK Tag (D6W5B) rabbit monoclonal antibody (14,793; Cell Signaling, USA; 1:50), as previously reported (Harms et al., 2015). The relative quantity of the immunoprecipitated factor was calculated by qPCR.

### Statistical analysis

All results are presented as the mean ± S.E.M, based on at least three independent experiments for each treatment. Statistical significance of differences between means was assessed by performing an unpaired Student's *t*-test and *P* < 0.05 or less was considered significant.

## Results

### cDNA sequence, genome structure, protein-encoding ability, and expression level of lncRNA-Six1

**Figure 1:**
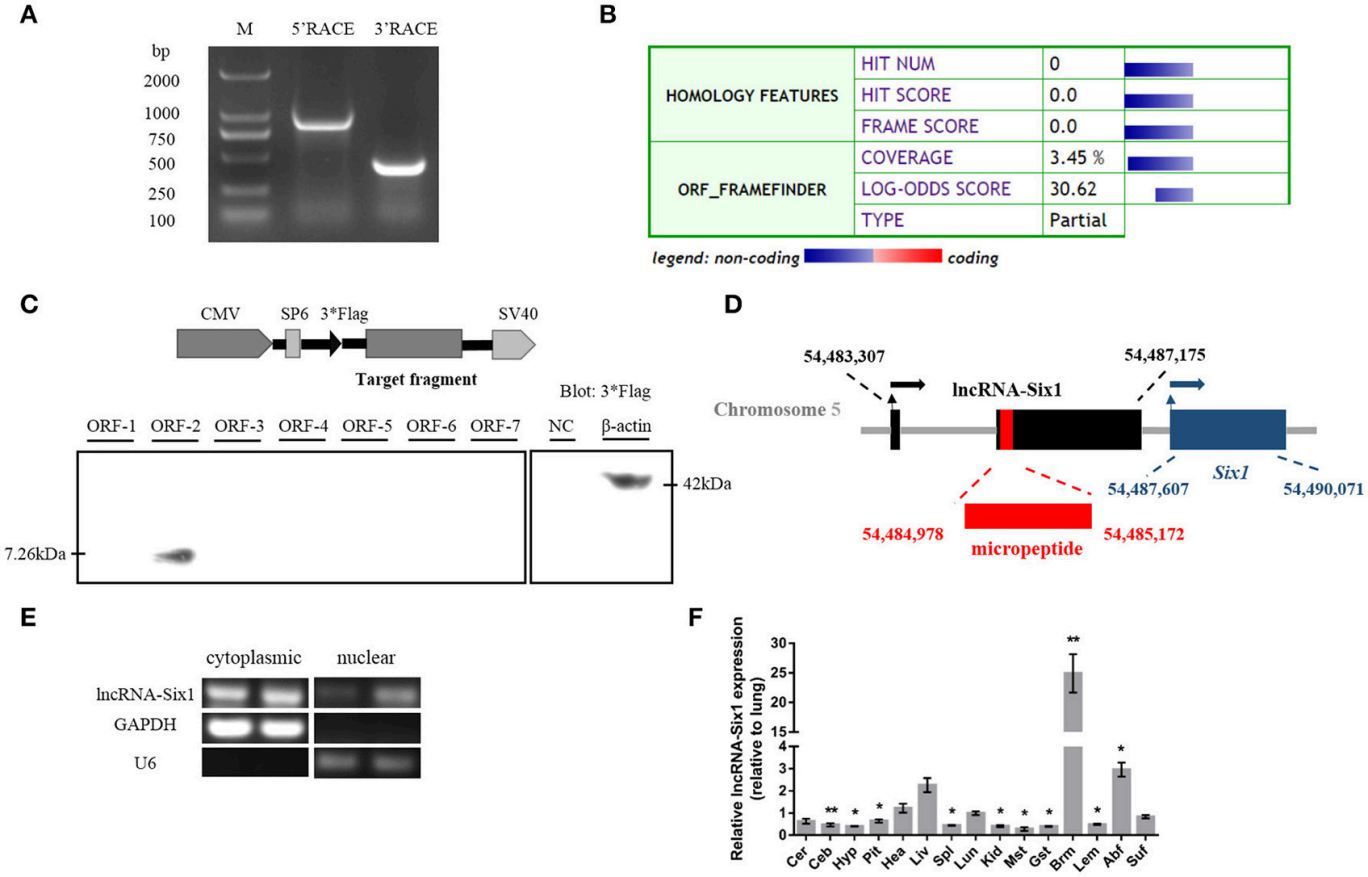
**Identification of lncRNA-Six1. (A)** Results of 5′ RACE and 3′ RACE. M, DL2000 Marker; 5′ RACE product, 806 bp; 3′ RACE product, 365 bp. **(B)** Analysis obtained from the Coding Potential Calculator (http://cpc.cbi.pku.edu.cn/) based on evolutionary conservation and ORF attributes. **(C)** Western blot analysis of the coding ability of lncRNA-Six1. The possible ORF of lncRNA-Six1 was cloned into the eukaryotic expression vector pSDS-20218 with a 3^*^Flag tag. Untransfected DF-1 cells were used as a negative control (NC) and DF-1 cells transfected with β-actin were used as a positive control. The upper panel shows the model target fragment in the pSDS vector. The lower left panel is the blot of ORFs 1–7, and the lower right panel is the blot of the NC and β-actin. **(D)** A schematic image of the locus for lncRNA-Six1 (black), micropeptide (red) and *Six1* (blue). Coordinates are listed according to the Gallus_gallus-5.0 reference Annotation Release 103. Thin arrows represent the transcription start site. Thick arrows represent the direction of transcription. **(E)** RT-PCR detection of lncRNA-Six1 in the cytoplasmic and nuclear fractions of chicken primary myoblasts. GAPDH and U6 serve as cytoplasmic and nuclear localization controls, respectively. **(F)** XH chicken tissue expression profiles of lncRNA-Six1. The horizontal axis and vertical axis indicate different tissues and their relative expression values (mean ± S.E.M). Cer, cerebrum; Ceb, cerebellum; Hyp, hypothalamus; Pit, pituitary; Hea, heart; Liv, liver; Spl, spleen; Lun, lung; Kid, kidney; Mst, muscular stomach; Gst, glandular stomach; Brm, breast muscle; Lem, leg muscle; Abf, abdominal fat; Suf, subcutaneous fat. In panel **(F)**, the results are shown as the mean ± S.E.M. and the data were representative of three independent assays from six female XingHua chickens. Statistical significance of differences between means was assessed using an unpaired Student's *t*-test. (^*^ *P* < 0.05; ^**^ *P* < 0.01.)

We identified the 5′ and 3′ ends of lncRNA-Six1 by RACE analysis (Figure 1A and Supplementary Data 1). Predictions of the identity of lncRNA-Six1 by the Coding Potential Calculator suggested a low coding potential and low evolutionary conservation consistent with a non-coding RNA (Kong et al., 2007; Figure 1B). In order to verify this prediction, we analyzed the coding ability of seven potential ORFs of lncRNA-Six1 by Western blot. We found that only ORF-2 generated a micropeptide (7.26 kDa), suggestive that lncRNA-Six1 is an lncRNA with low protein-encoding potential (Figure 1C and Supplementary Data 2). The National Center for Biotechnology Information's Basic Local Alignment Search Tool (BLAST) showed that the lncRNA-Six1 was 2,350 bp long, located on chromosome 5 and spanned from 54,483,370 to 54,487,175, and comprised two exons. The micropeptide was also located at chromosome 5 from 54,484,978 to 54,485,172, and the 3′ end of lncRNA-Six1 was separated from *Six1* by 432 bp (Figure 1D). We also investigated the intracellular localization of lncRNA-Six1, the RT-PCR results confirmed that lncRNA-Six1 is an RNA molecule present in the cytoplasm and nucleus (Figure 1E).

We measured lncRNA-Six1 expression from different tissues from 7-week-old XH chickens using RT-qPCR. LncRNA-Six1 was expressed in all 15 tissues, and breast muscle had the highest levels (Figure 1F). This suggested that lncRNA-Six1 may function in chicken skeletal muscle development.

### lncRNA-Six1 promotes cell proliferation and induces cell division

**Figure 2:**
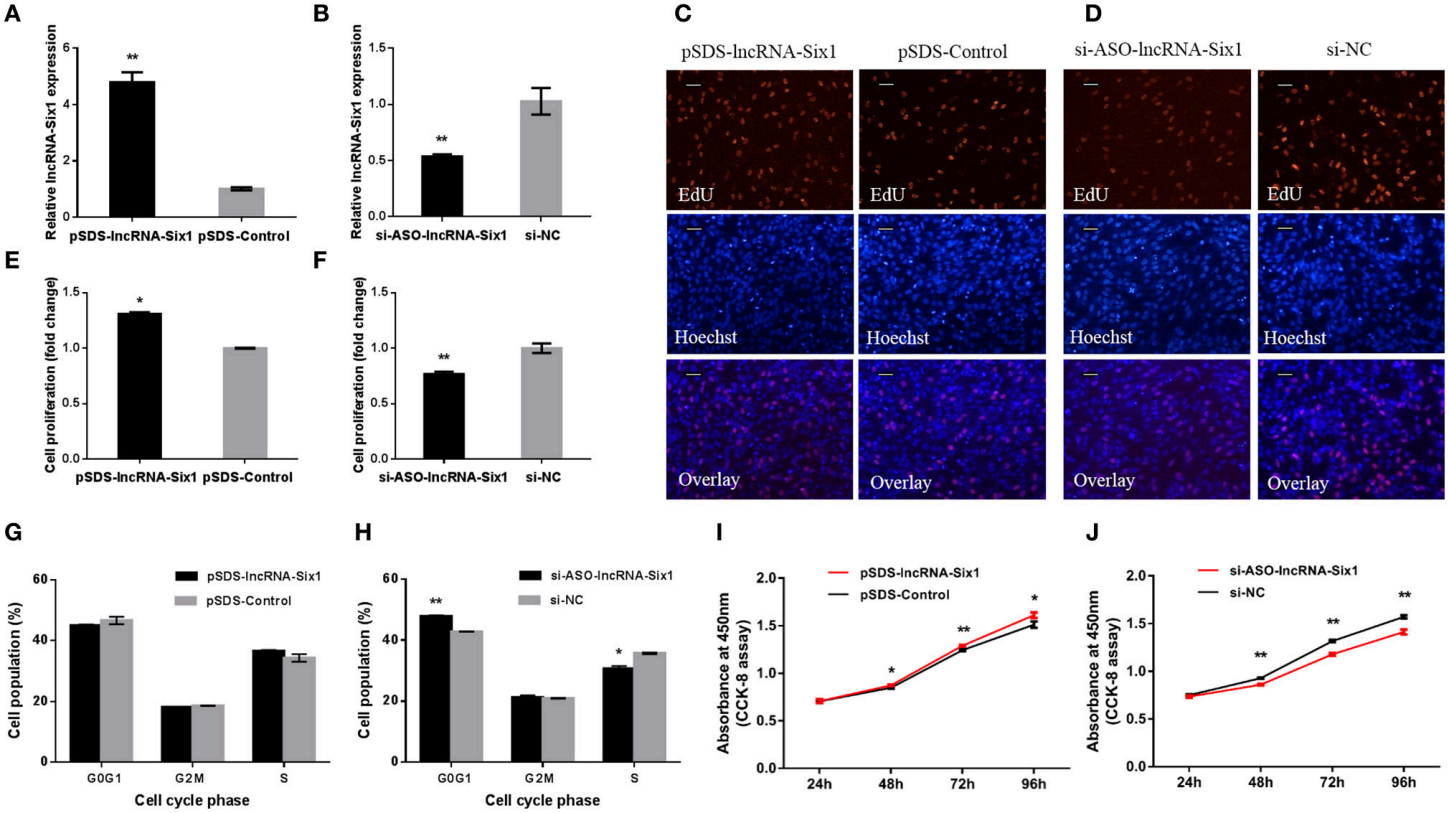
**lncRNA-Six1 promotes cell proliferation and induces cell division. (A,B)** lncRNA-Six1 relative expression determined by RT-qPCR in DF-1 cells after transfection with the listed nucleic acids. **(C,D)** EdU proliferation assays for the cells in **(A,B)** 48 h after transfection, Scale bar, 5 μm. **(E,F)** the numbers of proliferative cells were also counted. **(G,H)** Cell cycle analysis of DF-1 cells 48 h after transfection using propidium iodide staining for DNA content. **(I,J)** DF-1 cell growth curves following transfection of the indicated plasmids over time. In all panels, data are presented as the mean ± S.E.M. of three biological replicates with unpaired Student's *t*-test. (^*^ *P* < 0.05; ^**^ *P* < 0.01) vs. NC, negative control.

To unveil functions for lncRNA-Six1, we performed lncRNA-Six1 overexpression and knockdown experiments to assess its role in cell proliferation and viability. The plasmid pSDS-lncRNA-Six1 was constructed for lncRNA-Six1 overexpression, and siRNA and ASO for lncRNA-Six1 knockdown (si-ASO-lncRNA-Six1) were designed and synthesized. After 48 h of transfection, the relative RNA expressions of lncRNA-Six1 were detected (Figures 2A,B). LncRNA-Six1 overexpression significantly promoted cell proliferation, as judged by EdU incorporation (Figures 2C,E). Conversely, proliferation was significantly inhibited after lncRNA-Six1 knockdown (Figures 2D,F). LncRNA-Six1 overexpression also resulted in a slight decrease in the number of G0/G1 cells, and a slightly increase in the number S phase cells. LncRNA-Six1 knockdown reversed this trend, and G0/G1 cells increased significantly and S phase cells decreased significantly (Figures 2G,H). In addition, we also used the CCK-8 assay and found that lncRNA-Six1 overexpression promoted an increase in cell number. However, with lncRNA-Six1 interference, the opposite was true (Figures 2I,J). Together, these data were indicative that lncRNA-Six1 has a positive regulatory effect on cell growth and proliferation, and induces cell division.

### *Six1* is a targeted regulatory gene of lncRNA-Six1 in chicken

**Figure 3:**
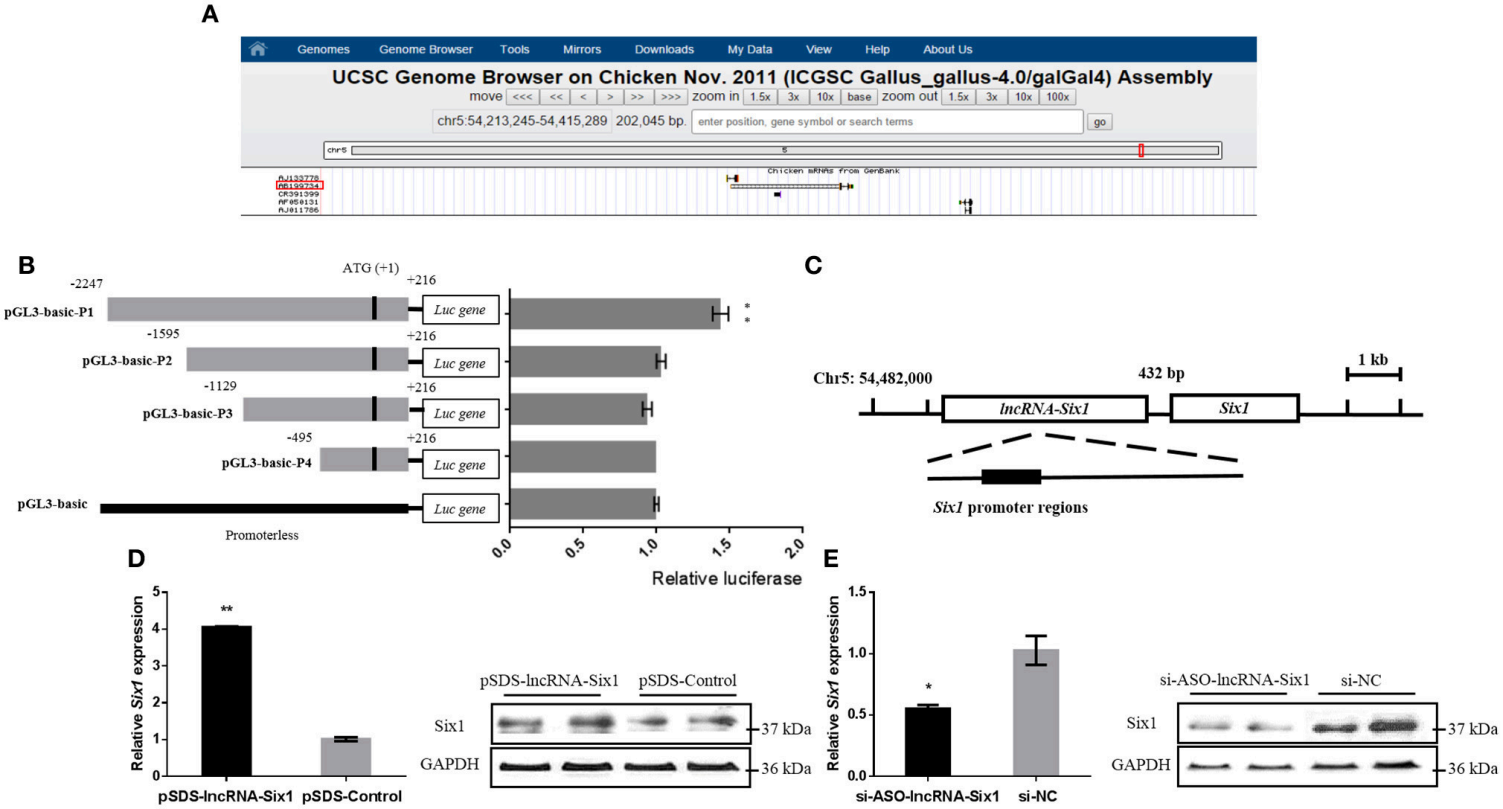
***Six1* is a targeted *cis*-regulatory gene of lncRNA-Six1. (A)** UCSC Genome Browser on Chicken Nov. 2011 (ICGSC Gallus_gallus-4.0/galGal4) assembly showing the positional relationship between lncRNA-Six1 and *Six1*. **(B)** Dual-luciferase reporter assay using the clones' upstream regions of the chicken *Six1* promoter. **(C)** Model of lncRNA-Six1 *cis*-regulated *Six1*. **(D)** The mRNA and protein expression levels of *Six1* from pSDS-lncRNA-Six1 transfected DF-1 cells. **(E)** Knockdown of the *Six1* gene in transfected DF-1 cells. In all panels, data are presented as means ± S.E.M. of three independent experiments with unpaired Student's *t*-test. (^*^ *P* < 0.05; ^**^ *P* < 0.01.) vs. NC, negative control.

In order to further understand the molecular mechanism of lncRNA-Six1, we predicted its *cis*-regulated target genes on the UCSC Genome Browser (http://genome.ucsc.edu/) (The differentially expressed genes at 100 Kb upstream or downstream of differentially expressed lncRNAs were selected as the potential *cis* target genes of lncRNAs) (Li et al., 2017; Figure 3A). The results showed that the lncRNA-Six1 is located upstream of *Six1*, which suggests that *Six1* may be a potential *cis*-regulatory target gene for lncRNA-Six1.

To investigate whether lncRNA-Six1 targeted regulate *Six1*, we cloned individual segments from the *Six1* promoter in front of firefly luciferase. These constructs were used to transiently transfect DF-1 cells to determine the region required for *Six1* promoter activity. Deletions extending from −1595 to +216, −1129 to +216, and −495 to +216 showed no significant difference in luciferase activities compared with the pGL3-basic control group. However, a significant increase of luciferase activity was observed with the longest construct (−2247 to +216) when compared with the region from −1595 to +216. This was indicative that an essential region of the *Six1* promoter was located at −2247 to −1595 bp (Figure 3B). Interestingly, the 3′ end of lncRNA-Six1 was separated from *Six1* by 432 bp and the whole RNA overlaps the key promoter regions of *Six1* (Figure 3C). This was suggestive that this lncRNA is the regulatory factor of *Six1*.

More importantly, after overexpression of lncRNA-Six1, the mRNA and protein levels of *Six1* increased, while inhibition of lncRNA-Six1 down-regulated *Six1* mRNA and protein (Figures 3D,E). Therefore, we confirmed that *Six1* is a direct target gene of lncRNA-Six1 in chickens.

### The micropeptide is required for lncRNA-Six1 to act *in cis*

**Figure 4:**
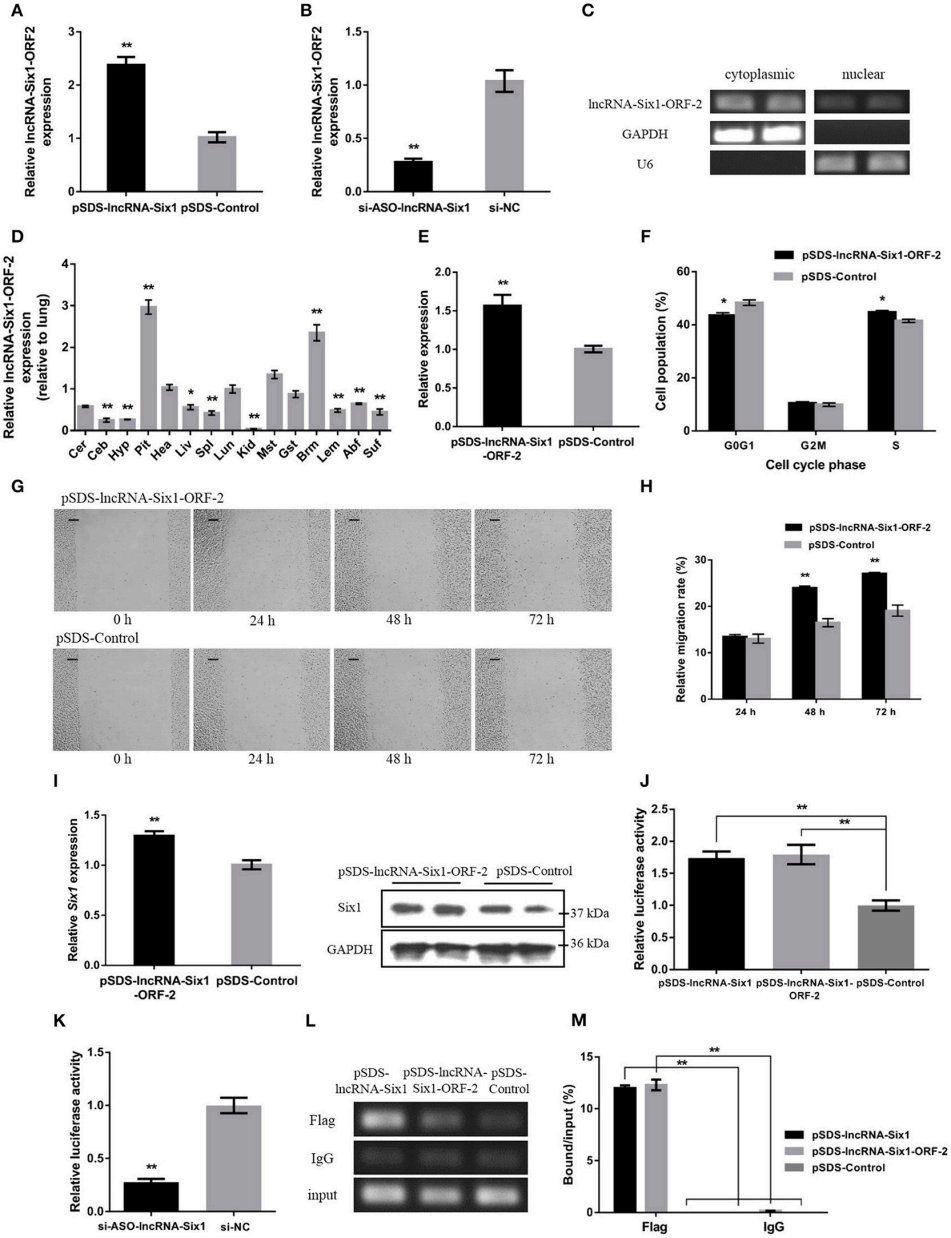
**The micropeptide is a key factor required for lncRNA-Six1 to act *in cis*. (A,B)** The mRNA expression levels of lncRNA-Six1-ORF-2 from pSDS-lncRNA-Six1 or si-ASO-lncRNA-Six1 transfected DF-1 cells. **(C)** RT-PCR detection of lncRNA-Six1-ORF-2 in the cytoplasmic and nuclear fractions of chicken primary myoblast. **(D)** Tissue expression profiles of the lncRNA-Six1-ORF-2 in XH chickens. The abbreviations for the tissue names are as described in Figure 1. **(E)** LncRNA-Six1-ORF-2 mRNA expression levels after overexpressing lncRNA-Six1-ORF-2 in DF-1 cells. **(F)** Cell cycle analysis of DF-1 cells after lncRNA-Six1-ORF-2 overexpression. **(G)** Representative images of the scratch-wound assay at 0, 24, 48, and 72 h after overexpression of the lncRNA-Six1-ORF-2 in DF-1 cells. Scale bar, 37.5 μm. **(H)** Quantification of the scratch-wound assay at 24, 48, and 72 h after overexpression of the lncRNA-Six1-ORF-2. **(I)** The mRNA and protein expression levels of *Six1* from pSDS- lncRNA-Six1-ORF-2 transfected DF-1 cells. **(J, K)** Dual-luciferase reporter assay of the *Six1* promoter activity after overexpression of lncRNA-Six1 and lncRNA-Six1-ORF-2, or interference of lncRNA-Six1. **(L)** Chromatin immunoprecipitation PCR analysis of the binding capacity of lncRNA-Six1 and the micropeptide to the *Six1* promoter region. **(M)** Chromatin immunoprecipitation qPCR analysis of the binding capacity of lncRNA-Six1 and the micropeptide to the *Six1* promoter region. In all panels, results are expressed as the mean ± S.E.M. of three independent experiments with unpaired Student's *t*-test. (^*^ *P* < 0.05; ^**^ *P* < 0.01.) vs. NC, negative control.

To further investigate whether the micropeptide was involved in the *cis*-regulation of *Six1*, the relative mRNA expression of the lncRNA-Six1-ORF-2 was detected after overexpression or interference of lncRNA-Six1 (Figures 4A,B). The intracellular localization and tissue expression profiles of lncRNA-Six1-ORF-2 were also analyzed (Figures 4C,D). The results showed that lncRNA-Six1-ORF-2 was present in the cytoplasm and nucleus, and highly expressed in breast muscle. Overexpression of lncRNA-Six1-ORF-2 was performed to study its physiological effects (Figure 4E). A cell cycle analysis after overexpression of lncRNA-Six1-ORF-2 was carried out (Figure 4F). Moreover, we also used the scratch-wound assay and found that lncRNA-Six1-ORF-2 overexpression promoted cell proliferation and migration (Figures 4G,H).

The mRNA and protein levels of *Six1* were significantly increased after overexpression of lncRNA-Six1-ORF-2 (Figure 4I). Meanwhile, a dual-luciferase reporter assay was developed to measure the *Six1* promoter activity, after co-transfection with pSDS-lncRNA-Six1, pSDS-lncRNA-Six1-ORF-2, and si-ASO-lncRNA-Six1 with pGL3-basic-P1 (Figures 4J,K). We found that the relative luciferase activity was significantly increased with the overexpression of lncRNA-Six1 or lncRNA-Six1-ORF-2, while the knockdown of lncRNA-Six1 caused the relative luciferase activity to be decreased significantly. In the chromatin immunoprecipitation assay, it was found also that the micropeptide was enriched by the *Six1* promoter region (Figures 4L,M). These results indicate that the micropeptide may be a key factor required for lncRNA-Six1 to act *in cis*.

### *Six1* promotes chicken skeletal muscle growth

**Figure 5:**
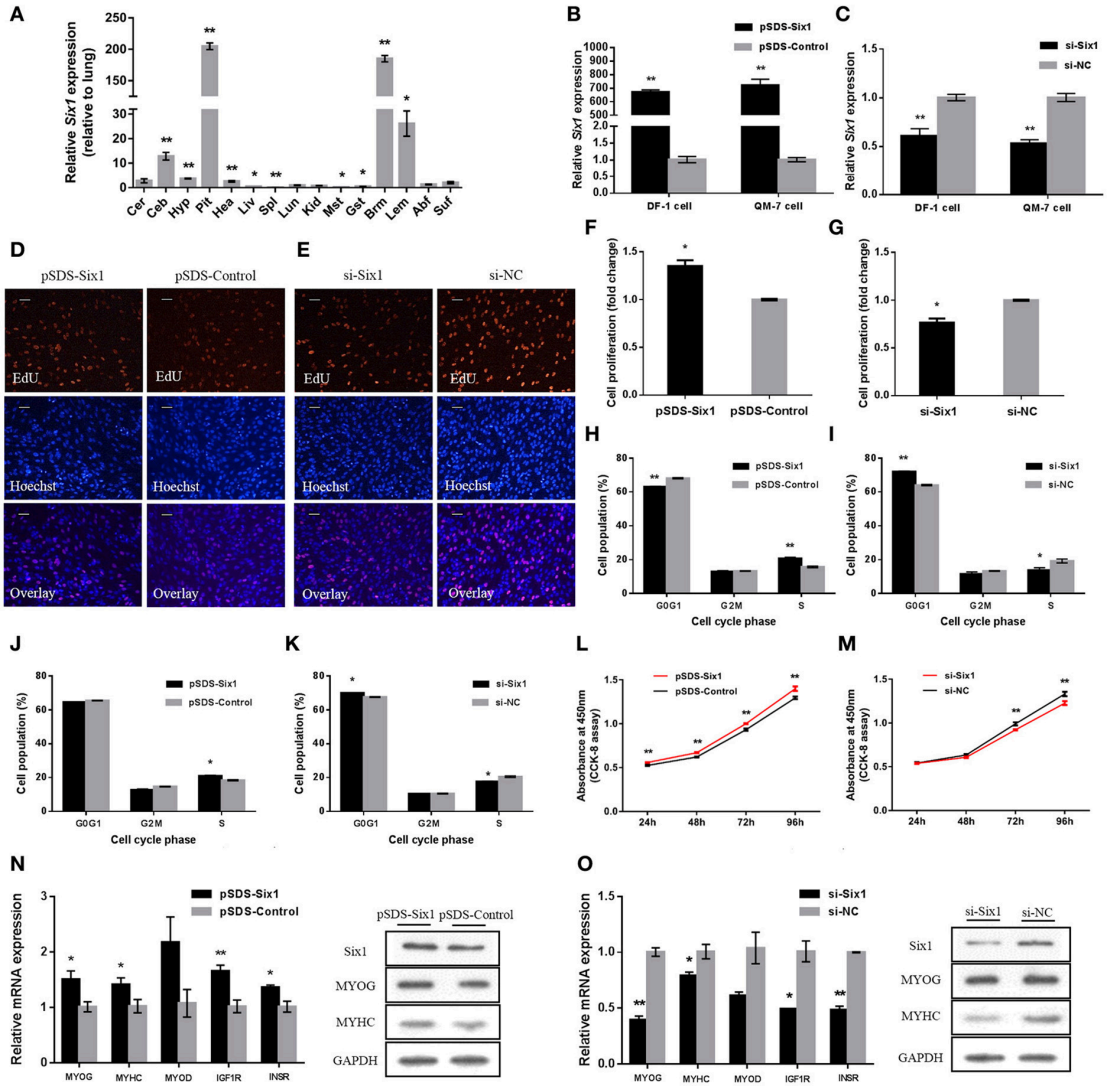
***Six1* promotes the growth of chicken skeletal muscle. (A)** Tissue expression profiles of *Six1* in XH chickens. The abbreviations for the tissue names are as described in Figure 1. **(B,C)** *Six1* mRNA levels in **(B)** transfected and **(C)** knockdown DF-1 and QM-7 cells. **(D,E)** Proliferation of transfected DF-1 cells was assessed by EdU incorporation. Scale bar, 5μm. **(F,G)** Proliferation rates of DF-1 cells transfected with pSDS-Six1 and with si-Six1. **(H–K)** Both DF-1 and QM-7 cells, respectively, were collected for cell cycle analysis 48h after transfection. **(L,M)** Cell growth was measured following the transfection of pSDS-Six1 and si-Six1. **(N,O)** mRNA and protein levels of some related genes induced by *Six1* overexpression and siRNA interference in DF-1 cells. In all panels, results are expressed as the mean ± S.E.M. of three replicates with unpaired Student's *t*-test. (^*^ *P* < 0.05; ^**^ *P* < 0.01.) vs. NC, negative control.

We examined tissue expression profiles for *Six1* and found high expression in breast and leg muscle (Figure 5A). *Six1* overexpression and knockdown in DF-1 and QM-7 cells verified the positive regulatory function of *Six1* in myogenic proliferation (Figures 5B,C). In DF-1 cells, overexpression of *Six1* significantly promoted cell proliferation (Figures 5D,F). This also resulted in a significant decrease in the number of cells in G0/G1, and a significant increase in the number of S-phase cells (Figure 5H). However, cell proliferation was inhibited after interference of *Six1* expression (Figures 5E,G), and resulted in a larger number of G0/G1 cells and fewer S phase cells (Figure 5I).

We found similar results for transfected QM-7 myoblasts. *Six1* overexpression significantly reduced the number of cells that progressed to G0/G1 and increased the number of S phase cells (Figure 5J). These observations coincided with enhanced cell viability (Figure 5L). Conversely, *Six1* knockdown significantly inhibited myoblast proliferation and reduced cell viability (Figures 5K,M).

To identify the role of *Six1* in the chicken skeletal muscle growth, the mRNA expression level of growth-related genes, such as *MYOG, MYHC, MYOD, IGF1R*, and *INSR*, were detected by qPCR after overexpression or knockdown of *Six1*. Furthermore, the protein levels of *MYOG* and *MYHC* were also detected by Western blot. The results showed that overexpression of *Six1* could promote the upregulation of growth-related genes (Figure 5N). In contrast, growth-related gene expression was inhibited after interference with *Six1* (Figure 5O). This data demonstrated that *Six1* promoted the expression of muscle-growth-related genes and further induced chicken muscle growth.

## Discussion

With the development of genome research, it has been found that the transcription of the animal genome is not only ubiquitous, but also incredibly complex. In the past, lncRNA was considered to be part of the “noise” of the transcribed genome, without any function. However, there is growing evidence that lncRNAs can serve as versatile regulators of diverse aspects of biology by a variety of mechanisms (Rinn et al., 2007). LncRNAs are a class of RNA generally considered to be non-coding with low sequence conservation. However, for some lncRNAs, low protein-encoding potential have been shown. A minimum ORF length is generally used to discriminate between protein-encoding genes and lncRNAs (Karapetyan et al., 2013). For example, the FANTOM consortium originally considered the protein-encoding gene to have an ORF of at least 300 nucleotides (100 amino acids) (Okazaki et al., 2002), and this result is highly consistent with subsequent studies using more sophisticated methods of identification (Frith et al., 2006a,b). Another alternative approach to discriminating lncRNAs from protein-encoding genes is to assess putative ORFs for similarity to known proteins or protein domains, since such homology provides indirect evidence of function as an mRNA (Dinger et al., 2008). In the present study, we identified a lncRNA (lncRNA-Six1) that was differentially expressed between WRR and XH chickens. For this lncRNA, the evolutionary conservation was analyzed and ORF attributes were predicted by the Coding Potential Calculator. We found a micropeptide of about 7.26 kDa using Western bolt assays, which demonstrated that lncRNA-Six1 is an lncRNA. LncRNA-Six1 is present in the cytoplasm and nucleus, and showed a higher expression level in breast muscle.

There is evidence that lncRNAs function during muscle growth. A muscle-specific mouse lncRNA (linc-MD1) is a competitive endogenous RNA for miR-133 and miR-135 targets. This lncRNA controls the expression of *MEF2C* and *MAML1*, which are transcription factors that activate late-differentiation muscle genes; thus, affecting myoblast differentiation (Cesana et al., 2011). Moreover, Linc-YY1, a dynamically regulated factor during myogenesis *in vitro* and *in vivo*, was discovered from the promoter of the transcription factor Yin Yang 1 (*YY1*) gene (Zhou et al., 2015). The study found that Linc-YY1 can activate the gene expression of *YY1 in trans* by interacting with *YY1*; thus, altering myogenic differentiation and affecting the regeneration of impaired muscle. The *MyoD* upstream non-coding RNA (MUNC) is specifically expressed in skeletal muscle and can act directly or indirectly on multiple promoters to increase myogenic gene expression, affecting myoblast differentiation in myogenesis (Mueller et al., 2015). LncRNA-Six1 showed a higher expression level in breast muscle, which is suggestive that lncRNA-Six1 may have a role in chicken muscle growth.

LncRNAs are primarily *cis*-acting regulatory elements and have been described for numerous regulators, including Xist, lincRNA-p21, Kcnq1ot1, and ANRIL (Clemson et al., 1996; Pandey et al., 2008; Yap et al., 2010; Dimitrova et al., 2014). In our study, using an *in silico* analysis, we identified lncRNA-Six1 upstream of the *Six1* gene, which itself was also differentially expressed. Interestingly, lncRNA-Six1 was found to overlap the *Six1* promoter. Moreover, the relative luciferase activity was significantly increased with the overexpression of lncRNA-Six1, while the knockdown of lncRNA-Six1 caused the relative luciferase activity to be decreased significantly, suggesting it regulates via a *cis* mechanism.

Interestingly, recent studies have shown that lncRNAs could also form micropeptide by encoding short ORFs, thus, directly regulating a large variety of functions. Myoregulin (MLN), which is a micropeptide encoded by an annotated lncRNA, was discovered to inhibit the pump activity of sarcoplasmic reticulum Ca^2+^-ATPase (SERCA), affecting exercise performance and Ca^2+^ handling in muscle (Anderson et al., 2015). In mice, a peptide, DWORF (dwarf open reading frame), of 34 amino acids was discovered in a putative muscle-specific lncRNA. DWORF can enhance muscle performance by activating the same calcium pump, and may prove to be useful in improving the cardiac muscle function of mammals with heart disease (Nelson et al., 2016). Another recent study identified a small polypeptide (small regulatory polypeptide of amino acid response, SPAR) encoded by the lncRNA LINC00961, which can inhibit amino acid-induced mTORC1 activation and muscle regeneration in skeletal muscle (Matsumoto et al., 2017). In our study, we have identified a micropeptide encoded by lncRNA-Six1. LncRNA-Six1-ORF-2 was present in the cytoplasm and nucleus, and highly expressed in breast muscle. Overexpression of lncRNA-Six1-ORF-2 promoted cell proliferation and migration, and the micropeptide was enriched by the *Six1* promoter region. We speculate that this micropeptide plays an important role in lncRNA-Six1 *in cis* activity.

In vertebrates, *Six1* is a regulator of skeletal muscle development and its mRNA is localized to regions of myogenesis in mouse embryos (Oliver et al., 1995; Buckingham and Rigby, 2014). In zebrafish, the *Six1a* ortholog drives proliferation and differentiation of zebrafish embryonic muscle fiber precursor cells, and its knockdown results in apoptosis (O'Brien et al., 2014). *Six1* also plays a key role in the transcriptional regulation of the myogenic regulatory factor (MRF) gene family (Fougerousse et al., 2002; Grifone et al., 2005). For *in situ* hybridization, no *MyoD*- and *MyoG*-expressing cells are detected in *Six1*−/− limb buds at E11.5 (Laclef et al., 2003). During the differentiation of C2C12 cells, *Six1* was down-regulated and the same down-regulation in expression was shown in *Myf5, MyoD*, and *MyoG* (Wu et al., 2012). Moreover, the expression of *MyHCIIB* or *MyHCIIX* was never observed by immunohistochemistry in Sol fibers, when *Six1* could not be detected in peripheral nuclei of these fibers (Grifone et al., 2004). *IGF1R* and *INSR* are the insulin receptors and have been found to be involved in the regulation of animal growth. A study showed that *Six1, IGF1R*, and *INSR* are essential for the correct initiation and up-regulation of *SRY*, and affect mammalian gonad development (Eggers et al., 2014). Similarly, our results are indicative that *Six1* has a high expression level in chicken muscle tissue. *Six1* overexpression also significantly promoted proliferation of myoblast cell lines. Furthermore, overexpression of *Six1* could promote the expression of *MYOG, MYHC, MYOD, IGF1R*, and *INSR*. These genes were down-regulated with knockdown of *Six1*.

In this study, we have confirmed that lncRNA-Six1 locates in the upstream of *Six1* and overlaps the *Six1* proximal promoter. Overexpression or knockdown of lncRNA-Six1 promoted or inhibited, respectively, the mRNA and protein expression level of *Six1*. These data demonstrate that *Six1* is a direct *cis*-acting target of lncRNA-Six1. In addition, a micropeptide of about 7.26 kDa encoded by lncRNA-Six1 was found to be required for lncRNA-Six1 to act *in cis*. This micropeptide was enriched by the *Six1* promoter region to regulate *Six1* expression. Overexpression of lncRNA-Six1 or *Six1* promoted cell proliferation and cell division, while the opposite effects were observed after knockdown of lncRNA-Six1 or *Six1*. Moreover, overexpression or knockdown of *Six1* promoted or inhibited, respectively, the expression of muscle-growth-related genes. Taken together, the results of our study demonstrate that lncRNA-Six1 carries out *cis*-acting regulation of the protein-encoding *Six1* gene. A micropeptide encoded by lncRNA-Six1 binds with the promoter of *Six1* and activates *Six1* to promote cell proliferation and cell division, and induce the expression of muscle-growth-related genes in chicken.

## Author contributions

BC: Performed research, analyzed data, wrote paper. ZL: Analyzed data and participated in its design. MM: Constructed vectors. ZW and PH: Analyzed data. BA: Reviewed the manuscript. QN: Conceived of the study, and participated in its design and coordination. XZ: Participated in the design of the study.

## Funding

This research was supported by the Program for New Century Excellent Talents in University (NCET-13-0803), the Natural Scientific Foundation of China (31571269), the Foundation for High-level Talents in Higher Education in Guangdong, China.

### Conflict of interest statement

The authors declare that the research was conducted in the absence of any commercial or financial relationships that could be construed as a potential conflict of interest.
