# Supplementary material for: LncRNA-Six1 Encodes a Micropeptide to Activate Six1 in Cis and Is Involved in Cell Proliferation and Muscle Growth
Source: Front Physiol. 2017 Apr 20;8:230. doi: 10.3389/fphys.2017.00230 (PMC5397475; doi:10.3389/fphys.2017.00230)
Supplement: Supplementary file 1 [file DataSheet1.DOCX]

**Supplementary Information**

**LncRNA-Six1 Encodes a Micropeptide to Activate *Six1* in *Cis* and Involved in Cell Proliferation and Muscle Growth**

Bolin Cai^1, 2, 3^, Zhenhui Li^1, 2, 3^, Manting Ma^1, 2, 3^, Zhijun Wang^1, 2, 3^, Peigong Han^1, 2, 3^, Bahareldin Ali Abdalla^1, 2, 3^, Qinghua Nie^1, 2, 3*^, Xiquan Zhang^1, 2, 3^

**Running title**: LncRNA-Six1 regulates *Six1*

^1^Department of Animal Genetics, Breeding and Reproduction, College of Animal Science, South China Agricultural University, Guangzhou 510642, Guangdong, China

^2^Guangdong Provincial Key Lab of Agro-Animal Genomics and Molecular Breeding, and Key Laboratory of Chicken Genetics, Breeding and Reproduction, Ministry of Agriculture, Guangzhou 510642, Guangdong, China

^3^National-Local Joint Engineering Research Center for Livestock Breeding, Guangzhou 510642, Guangdong, China

**^*^Correspondence:**

Qinghua Nie

[nqinghua@scau.edu.cn](mailto:nqinghua@scau.edu.cn)

**Keywords:** lncRNA-Six1; *cis*-acting; micropeptide; *Six1*; muscle growth

**Supplementary Data S1:** The full-length sequence of lncRNA-Six1. Coordinates are listed according to the Gallus_gallus-5.0 reference Annotation Release 103. Bold represents the splice site of two exons.

**Gallus_gallus-5.0 (chr5: 54,483,370-54,483,509 and 54,484,966-54,487,175)**

GCCTGCTCCTGCAGCCCCGCTCGGGCCGGGTGAGCTCGTGAGAAGGGGGCAAAAAGCTGCAGCCGGCATCGCCTCGGGTCGTGGCAAATTGCGTTCTGCTGAGAAATGGGAAGAAAACGCGTGGGAACGGTCGGACACG**CA**ACAGCAAGTGGATGGGAAAGGAGCCCTCTCCAACACAAACCTCATTGACTAATGGGGCGGTTTTGGGTTTCGCCTTCGAAAGGTTCGGAAATCGGCAGCGTTCTGTTACCGCCCGCCCGGCTCCAGCCCAGAGCCCGCGGCTCTTATCGGACACCGCTCCCAGCGCTCCCTTCTGCTGCTCCTCTCTCCAGAAACCCGCACTTTAAAAACACATAGAGAGCTTTAGGTTTATTTGGTAGGCTTATTTCTAAGGCCGGAGGGGCAGAGGGGGGCCGCGTTTTCTATCTTTCCTATACAATTCTTTTTTTTTTTTTTTTTTTTCGGCGAAATCGGTGAAAAACGGCCGAGCGCTGCCCCGGCCCCATCCCCATCCCCGCTCCGCATCGAATAAACCCGCGGAGATGTGCGCTCCTGCGCAGGGATCGGGGCCGGGGTGAGCCCGACCCGTACGGCACAGAACCTCCCCGAGCCGCCCTGCCGCCGTTCGGCCGTTCGCTTCGAGCTCCGTTCCTTTGCTTTTATTCGAGCGCTTCGAAACCATTTCTCGTCTGAATTTATGGGGCTAAACGGAGCGTGAGGTTCGGAAATCCGTGCCGTTTTGCTTCTTTCGGGTGGTTTGAAGCGAAAACTTTCCGTTTGTTTTGTAGGAAAAGCAGCGGGTAACGCACGCCGCTGGGAGCGGCCCCGGCCGCAGCGCTGCCCGCGGGCGCAGCCCCGTTCGCTCCGCGGGCGCTGTGGGGCGCGGAGCGGCGCGGCCCCGGTGGGGGCTGAGCCCCAACGTTCGGGTTCGGCCTTGGGAAGGGACCGGCAGGGAGAAGCCGCTCTCGTTACAAACCAGGGCTCCTCACGGCCCCGCTTTGCGTTCTTCACCCCTTAGCCCTGAAGGGACGAACCCTCAGGTCCGTGCTTCCCAAACGTCTTGGAAAATTAGAGGAAGAGCGCATTGTAATAAAGGGAAAGGGACCGGGGAGCGCGTCCCGGGCTCGGTGCGGCCGTGGCACCGCTCCTGCGGGAGGCTCGGAGCCCACCGCTGTAATTCTGCGCACTTGCTCCAGACTTTAGGGTATTAGTTGGTGTTAGAGCCGGGTTTAAGGCTCCGTTCCGGTCCCCACGCAGCTGCCTTGTCAATAAGGATCTGTTCCAAGCAGACATTCCTTCTGGGAGGACATTGCAGCTCTTTAAGACTTCATCTGCAACATCACAAGCACTAAAATAAAATGGAAGGGAAAATTAGCAGACACGCGGTCACGGGACACGGCGGTGCCCGAGCGGTGCAGAGCGGACAGACGGGCGGCAGCGCCTTATTTAACCGCCCCGAGAAGAGAAATACGTGGGAAAAACAACCACCGGGGCACGGGGGGACGGCGGCGGGGCCTGCGGCCGGGGTGCGGCGGGAGGGACCCGGCCGGGTCCACCCGGAGGGTTCGGGGCCGGGTATCGGGTACCGGGATGGGGGCAGCGGGAGCACCGCCGGCCACAGCTCGGAGCCCTCCCCGGCCGGGCCGTGCGGAGCTCTCACGGCGGAATAAATCACCCCGCTCTCAGAGAAGACGAACAGGTTTTCAAGAGCTCCTCGATTGTTCTCAGAACCCACGGAGTGAGCCCAAACGCCGGCTGGAGGGTGCTCAAAGCGCAGCGCTCCGCTTATCTCAGCGCGTATAAACGCTGCCATAAAGACACCCTCATCCCCGCCCTTATCTCAGCGCATTGTCCCCATCTCCCAGCCCGCCCCGGCTCCCGACGGCAGCTTATCTCTGCTTGGGGAACCTAATTGCTTTCTAATTTTTGCAATAATTGCTTTGGAGCGAGGTGTGCAGATGAGGATTTGGGCGAATGAAAGCGCCGCCGCCGGCTTTTCTCTTCCCCCTTCCCCGGGGGCGGCTCCGAGGGCCGCTCTCTGCCGACCTGAGCGGTCCCGGCCCGGACCCCCCCCGGCTGAGGGGGAGGCGGAGAGGTGCGGGGGGGGCCGCGCCTCCGCTCTGCTATTCAGCGCGCGGCATCCGCTGTGCGGCCGAGCGGTATTTATTCATTCACGCCTGGAGTGAGAGTGATGGAAGACGGGACGAGGAGTGGAGTTTAGAGAATGGAGAAGAAAGAAAAGAAAGAAATGAAAGTATATGAAAGGAAATAAATGAAAGAGAGAATTAAAAGAAAAAAAGAAAAAAGAAAAAAAGGAAAAAAAGAAAAAAAAAGGAAAAAAGGAAAAAAA

**Supplementary Data S2:** The sequence of lncRNA-Six1-ORF-2. Coordinates are listed according to the Gallus_gallus-5.0 reference Annotation Release 103.

**Gallus_gallus-5.0 (chr5: 54,484,978-54,485,172)**

ATGGGAAAGGAGCCCTCTCCAACACAAACCTCATTGACTAATGGGGCGGTTTTGGGTTTCGCCTTCGAAAGGTTCGGAAATCGGCAGCGTTCTGTTACCGCCCGCCCGGCTCCAGCCCAGAGCCCGCGGCTCTTATCGGACACCGCTCCCAGCGCTCCCTTCTGCTGCTCCTCTCTCCAGAAACCCGCACTTTAA
